# Unveiling the key role of metal coordination mode and ligand's side groups on the performance of deep-red light-emitting electrochemical cell

**DOI:** 10.1038/s41598-024-67159-7

**Published:** 2024-07-11

**Authors:** Babak Nemati Bideh, Ahmad Sousaraei, Majid Moghadam

**Affiliations:** 1 Department of Inorganic Chemistry, Faculty of Chemistry and Petroleum Sciences, Bu-Ali Sina University, Hamedan, Iran; 2 Departamento de Química Inorgánica, Facultad de Ciencias, Universidad Autónoma de Madrid, 28049 Madrid, Spain; 3 Department of Chemistry, University of Isfahan, Isfahan, 81746−73441 Iran

**Keywords:** Optical materials and structures, Optical physics

## Abstract

Three novel deep-red to near-infrared (DR to NIR) emitters based on mononuclear and dinuclear ruthenium(II) complexes with bulky structures were presented herein. For the first time, the unusual effects of metal coordination mode on the electroluminescence properties of a binuclear emitter were investigated. Unexpectedly, the mononuclear complexes showed superior performance in deep-red light-emitting electrochemical cells (DR-LEC) compared to the dinuclear complex. Likewise, substituting various ancillary ligands improved the radiance and lifetime of devices by 2.5 and 1.5 times, respectively. To the best of our knowledge, the obtained efficiency is among the best reported to date for DR-LECs based on ruthenium polypyridyl complexes.

## Introduction

**Figure 1:**
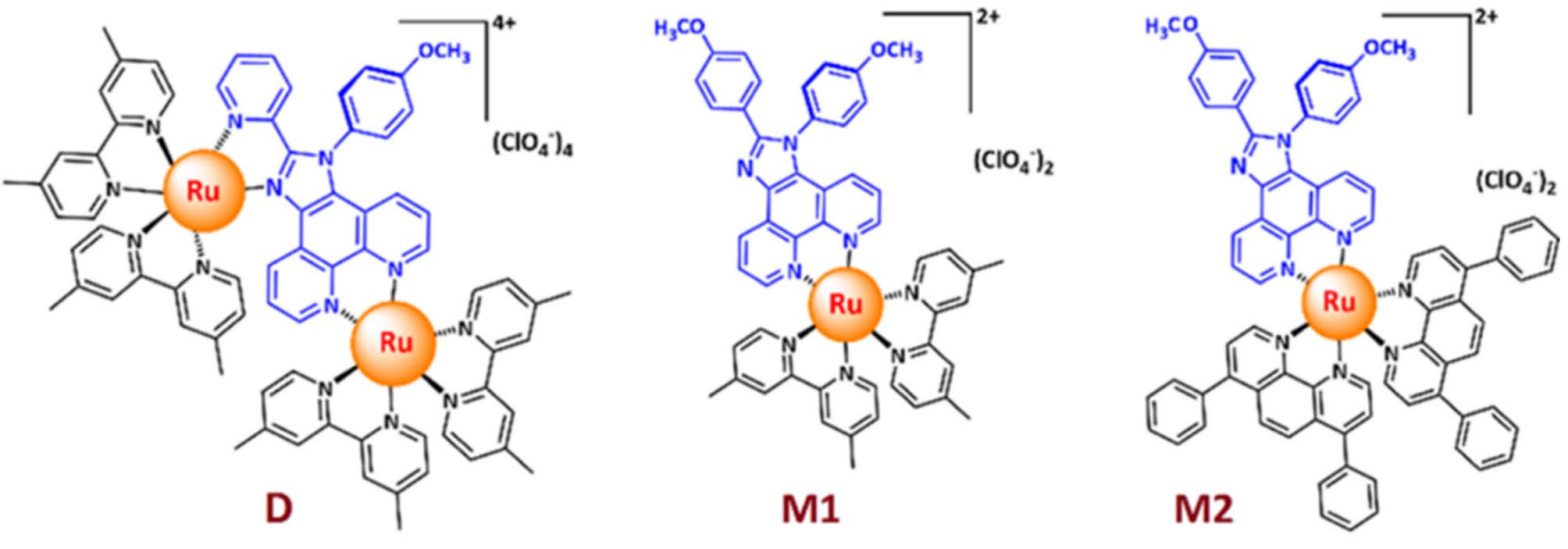
Chemical structures of mono- and dinuclear Ru(II) emitters.

Light-emitting electrochemical cells (LECs) are simple solution-processable thin film optoelectronic devices in which a single layer of an ionic luminescent material is sandwiched between two electrodes^1–5^. LECs possess some promising benefits over the most extensively explored organic light-emitting diodes (OLEDs), such as easily processed from solutions, compatibility with air-stable metal for the cathode material, and low-voltage driving. Such characteristics result from the working mechanism of LECs, in which ionic species migrate toward the respective electrodes under an external bias. Accumulation of ions in the vicinity of electrodes facilitates the p- and n-type doping processes within the emissive layer. Consequently, it reduces the driving voltage and sensitivity to the work function of the electrodes^1,4,5^. The various types of ionic materials such as ionic transition metal complexes (iTMCs), conjugated light-emitting polymers, organic small-molecules, quantum dots, and perovskite nanoparticles (NPs) have been successfully used as the emitter in LECs devices^4^. Amid, iTMCs present some superior properties compared to other kinds of materials. iTMCs are inherently ionic which exempts them from incorporating additional ion-transporting species. Meanwhile, iTMCs exhibit stable electrochemical red/ox properties. In addition, the phosphorescent nature of iTMCs emitters can harvest 100% of singlet and triplet excitons generated in an LEC device. In contrast, fluorescent materials (such as polymer and small molecules) are restricted to harvest only singlet excitons (25%)^4,6^. Therefore, phosphorescent materials show higher electroluminescence (EL) efficiency than fluorescence materials. Consequently, iTMCs have been largely investigated as emitters in the LEC devices in particular those emitting in the visible region of the spectrum^6,7^. However, iTMC-based LECs suffer from low stability and severe concentration quenching of emission^4^. It has been demonstrated that intramolecular π–π stacking interactions with the formation of the supramolecular cage, result in a significant stability improvement Ir(III)-based LECs^8^. Moreover, increasing the hydrophobicity of the active layer by attaching bulky side groups on the Ru(II) and Ir(III) complexes prevents the formation of quencher molecules through water substitution reactions, known as the limiting factors on the stability of LECs^6^. At the same time, the increase of the intermolecular distance in the active layer dramatically diminishes the concentration quenching of emission. In this regard, effective inter-complex distance can be achieved by introducing spacer (in multi-nuclear complexes) and bulky side groups in the structure of an emitter or adding an ionic liquid in the emissive layer^9,10^. On the other hand, it has been shown that multinuclearization of the Ru(II) and Ir(III) emitters increases the stability of the corresponding LECs by reducing the access of water molecules to the metal core^10,11^. According to the literature, these two factors have been less explored for the deep-red (DR) to near-infrared LECs (DR to NIR-LECs)^12^. Generally, all the reported DR to NIR-LECs suffer from low stability. Meanwhile, the reported deep-red and near-infrared LECs exhibited low or moderate device efficiencies due to the energy gap law^2,12^. Notwithstanding, the DR and NIR light-emitting devices have attracted great attention because of their wide potential applications, such as biosensors, phototherapy devices, optical signal detectors, night vision devices, etc.^2,9,12^. In this regard, DR to NIR-LECs are promising candidates due to their lower energy consumption, fabrication, and encapsulation costs. However, further improvement in DR/NIR-LECs device performance is required for practical applications. Considering, phenanthroimidazole (PI) ligands group possesses several advantages such as strong metal-binding moiety, variable bulky and rigid structure, good photophysical properties, and easy adjustment of their electronic properties by changing the imidazole moiety substitutions^13^. Furthermore, PI compounds are one of the key structures used in organic optoelectronics due to their photochemical, thermal, and morphological stability with better hole-transport capability^13^. The presence of electron-donating groups on the periphery of these ligands, as well as the construction of multinuclear complex by introducing a spacer in their ligand’s structures can improve in turn the efficiency and stability of the LECs^9,10^. It has been shown that the placement of methoxy groups on N^N ancillary ligands, especially in the case of iridium(III) complexes, can effectively increase the efficiency of electroluminescence^14,15^ .Furthermore, modifying the ligands by electron donating or withdrawing substitutions, and utilizing π-extended ancillary ligands leads to the color tuning from blue to near-infrared spectrum region^2,6,7^. Recently, it is demonstrated that the dinuclear phosphorescent complexes compared to their mononuclear counterparts have better efficiency in light-emitting devices due to the suppressed luminescence quenching in the emitter layers^9,10,16^. Inspired by these facts, we designed and synthesized three new mono- and dinuclear ruthenium(II) complexes based on bulky PI ligands with methoxy substitutions, namely M1, M2, and D (Fig. 1). Meanwhile, based on previous work^17^, various ancillary ligands (bathophen, dmbpy) were used to shift the emission of ruthenium complexes to longer wavelengths (DR region) compared with archetype [Ru(bpy)_3_]^2+^ complex. Concerning, the investigated EL properties of a few multinuclear complexes mostly focused on the role of the spacer groups that separate the emission centers^9,10,12^. According to detailed digging of the literature, it is found that the EL properties of multinuclear complexes without unique spacer groups with a relative small size have rarely been studied^9,10^.

The innovative aspect of the designed dinuclear complex D is its relatively small size of structure without a spacer group. Notably, in the phenanthreneimidazole material structure, there can be two N^N metal binding sites including imidazole-pyridine and phenanthroline moiety. Previously, some mononuclear complexes have been synthesized based on both bidentate PI ligand types for use as emitters in LEC applications^15,17,18^. However, in our newly designed ligand, both binding sites are active, and based on this, a new binuclear ruthenium(II), D, was synthesis and used as a DR-NIR emitter in LEC. Furthermore, we investigate the effect of occupation of both N^N coordination sites of PI ligand on the emission properties of ruthenium complex compared with two similar bulky mononuclear complexes.

## Results and discussion

### Synthesis and characterization

The full synthesis processes and characterization data for ligands and complexes are reported in the ESI. Three complexes were structurally characterized by TOF mass, ^1^H NMR, ^13^C NMR data, and elemental analysis that were consistent with the proposed structures of the complexes (see the ESI).

### Photophysical characterizations

**Figure 2:**
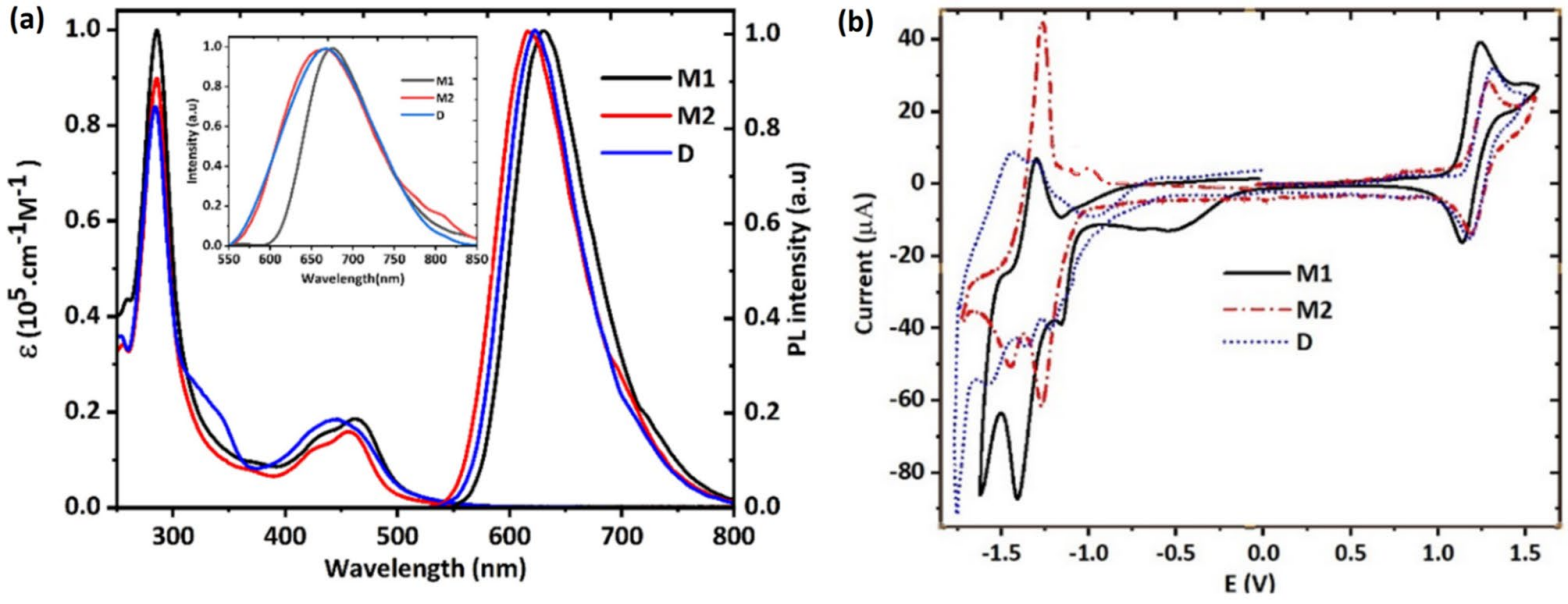
(**a**) UV–vis absorption and emission spectra of M1, 2 and D in ACN (1 × 10^–5^ M). Inset: the emission spectra of M1,2 and D in thin films. (**b**) Cyclic voltammograms of complexes M1, 2 and D in ACN solution at scan rate of 100 mV/s vs Ag/AgCl.

Figure 2a shows the absorption and photoluminescence (PL) spectra of complexes diluted in acetonitrile. Detailed photophysical characteristics are summarized in Table 1. The intense absorption bands in the ultraviolet region below 300 nm are attributed to the spin-allowed ligand-centred π–π* transitions. The relatively weak and broad absorption bands in the lower energy (400–500 nm) can be ascribed to the spin-allowed metal-to-ligand charge transfer (^1^MLCT) transition, whereas its broadening and asymmetric shape can be explained by an overlapping dπ → π*(dmbpy) and dπ → π*(PI) transitions. The additional shoulder for D around 330 nm is attributed to the intra-ligand transition from the bidentate PI ligand^19^.

**Table 1:** UV/Vis, emission, and redox properties of M1,2 and D.

Com	Absorbance^a^ ${\lambda }_{max}\left[nm\right] \left(log\varepsilon \right)$		Emission ${\lambda }_{max}[nm](\varphi \%)$		Ru(II/III) Oxi.^d^	Ligand Red^d^	E_HOM_	E_LUMO_	E_opt_, _gap_^e^
	Ligand transitions	MLCT	Solution^a,b^	Film^c^	E_1/2_($\Delta E$)(V)	E_red_ (V)			
M1	258 (4.64), 285 (4.99)	464 (4.27)	630 (11)	670 (16)	1.21 (0.089)	− 1.18 ^f^	− 5.58	− 3.36	2.22
M2	257 (4.53), 284 (4.95)	457 (4.21)	620 (9.3)	665 (15)	1.25 (0.087)	− 1.23 ^f^	− 5.60	− 3.31	2.29
D	252 (4.56), 283 (4.93)	448 (4.27)	623 (1.2 >)	668 (–)	1.22 (0.095)	− 1.22 ^f^	− 5.59	− 3.34	2.25
Ru(bpy)_3_^2+^	245 (4.4), 290 (4.91)	448 (4.17)	617 (9.5)	645 (12)	1.29 (0.079)	− 1.31	− 5.68	− 3.08	2.60

^a^In degassed CH_3_CN solutions (10^−5^ M) at 298 K.

^b^The $\varphi ($PLQY) were calculated by comparison with [Ru(bpy)_3_]^2+^ ($\varphi$_std_ = 0.095) in acetonitrile solution at room temperature.

^c^Films were made from complex: IL mixture (4:1) on glass substrates.

^d^Versus Ag/AgCl.

^e^Optical band gap.

^f^Irreversible peak.

The MLCT bands of these complexes are red-shifted compared with that of [Ru(bpy)_3_]^2+^, which may suggest whether an additional destabilization of the HOMOs of the complexes M1,2 and D due to the higher electron-donating nature of dmbpy and bathophen with respect to bpy ligands or stabilization of LUMOs that induced by π-extended PI ligand^16^. Upon photoexcitation at the MLCT band (at 450 nm) all complexes mainly emit in the red region centred at 630, 620, and 623 nm, respectively (see Table 1).

In addition, a PL quantum yield (PLQY) of 11% was determined for M1, which is higher than that of [Ru(bipy)_3_]^2+^ (9.5%) and is among the best-reported values for ruthenium polypyridil complexes. Furthermore, compared with mononuclear complexes of M1 and M2, dinuclear complex, D, depicted a significant decrease in PLQY (1.2% >), while usually phenanthroimidazole-based dinuclear complexes with spacer and their mononuclear analogs showed similar PLQY in solution^16^. This is attributed to the severe self-quenching of emission due to decreasing the internuclear distance^20^.

To evaluate the potential use of the complexes in LECs, the emission properties of the complexes were characterized in thin films of mixed Ru(II) complexes and ionic liquid of (ratio of 4:1, iTMC:IL, IL: [BMIM^+^(PF_6_)^−^], the same composition as used in the LEC fabrication). As shown in the inset of Fig. 2a, PL spectra of thin films exhibit similar shapes with respect to the solution emission and their emission maxima follow the same trend observed in the solution. However, in the thin films, complexes M1, M2, and D illustrate deep-red emission peaking at 670, 665, and 668 nm, respectively, which are red-shifted by over 40 nm compared to that in the solution. This red shift was attributed to the polarization effect in which the molecular orbitals of the emitters are affected by the ionic environment in the solid state or inter-complex π–π stacking interactions^21^. As shown in Table 1, the PLQYs of the complexes M1 and M2 have increased in the thin films compared to the solution due to decreasing the self-quenching of the emission in the thin films, owing to the increasing inter-complex distance by the addition of ionic liquid^15^. Furthermore, in the thin films, the PLQYs of the mononuclear complexes (M1, M2) are higher than that of [Ru(bpy)_3_]^2+^, even though their PLQYs in the solution are slightly different, demonstrating that presumably the bulky phenyl groups at the PI ligands significantly suppress the phosphorescence concentration quenching^22^.

### Electrochemical characterizations

In the operational mechanism of iTMC-LECs, the transport of electrons and holes takes place through consecutive oxidation and reduction of the metal complex during device operation. Therefore, the redox properties of the complexes are well reflected in the performance of their LECs. Hence, the cyclic voltammetry (CV) was carried out on M1, 2 and D in deaerated acetonitrile solution to assess electrochemical reversibility. The data recorded for M1, 2, and D were plotted (Fig. 2b), and the redox potentials are listed in Table 1. All complexes showed one mono-electron reversible oxidation peak and three quasi-reversible reduction peaks. The oxidation process is ascribed to the metal-centered oxidation that forms Ru(III) species, while the reduction processes correspond to the polypyridyl ligands, as suggested by previous literature findings^11,12,16^. In this regard, the lowest negative potential is attributed to the reduction of PI ligand^11^, which reflects a stabilization of the π* orbital of the PI ligand presumably due to its extensive π-conjugation system. The presence of reversible redox processes indicates the good electrochemical stability of mono and binuclear complexes, indicating that both holes and electrons could be transported well, which is beneficial to achieve efficient and stable iTMC-LECs^10,15^. All complexes depict somewhat similar redox properties, with nearly identical oxidation and reduction potentials and redox gaps (see Table 1). However, complex M1 exhibits a reversible oxidation peak at 1.21 V versus Ag/AgCl, which is slightly lower than that of complex M2 (1.25 V) and indicates the higher electron donation nature of dmbpy than bathophen. In addition, a significant anodic shift is observed for all complexes compared with [Ru(bpy)_3_]^2+^, which is attributed to the presence of electron-donor methyl and methoxy groups on the periphery of ligands. In addition, a single oxidation peak for D was observed within the potential window up to + 1.8 V despite there being two various Ru(II) centers in D. The subsequent oxidation occurs at higher potential (E_average_ = 2.35 V, see Fig. S9, ESI) in an irreversible manner, due to the electronic communication between two metal centers through π-conjugated, bis-bidentate PI ligand, which indicates that the efficiency issues might rise in D-based LEC device^11^. According to the calculated frontier orbital energy, the electron-donor groups and π-extended ligands in the complexes scaffold resulted in reduced energy gaps compared with archetype complex, [Ru(bpy)_3_]^2+^, through the stabilization of the LUMO and destabilization of the HOMO levels (see Table 1).

### Electroluminescent properties

**Figure 3:**
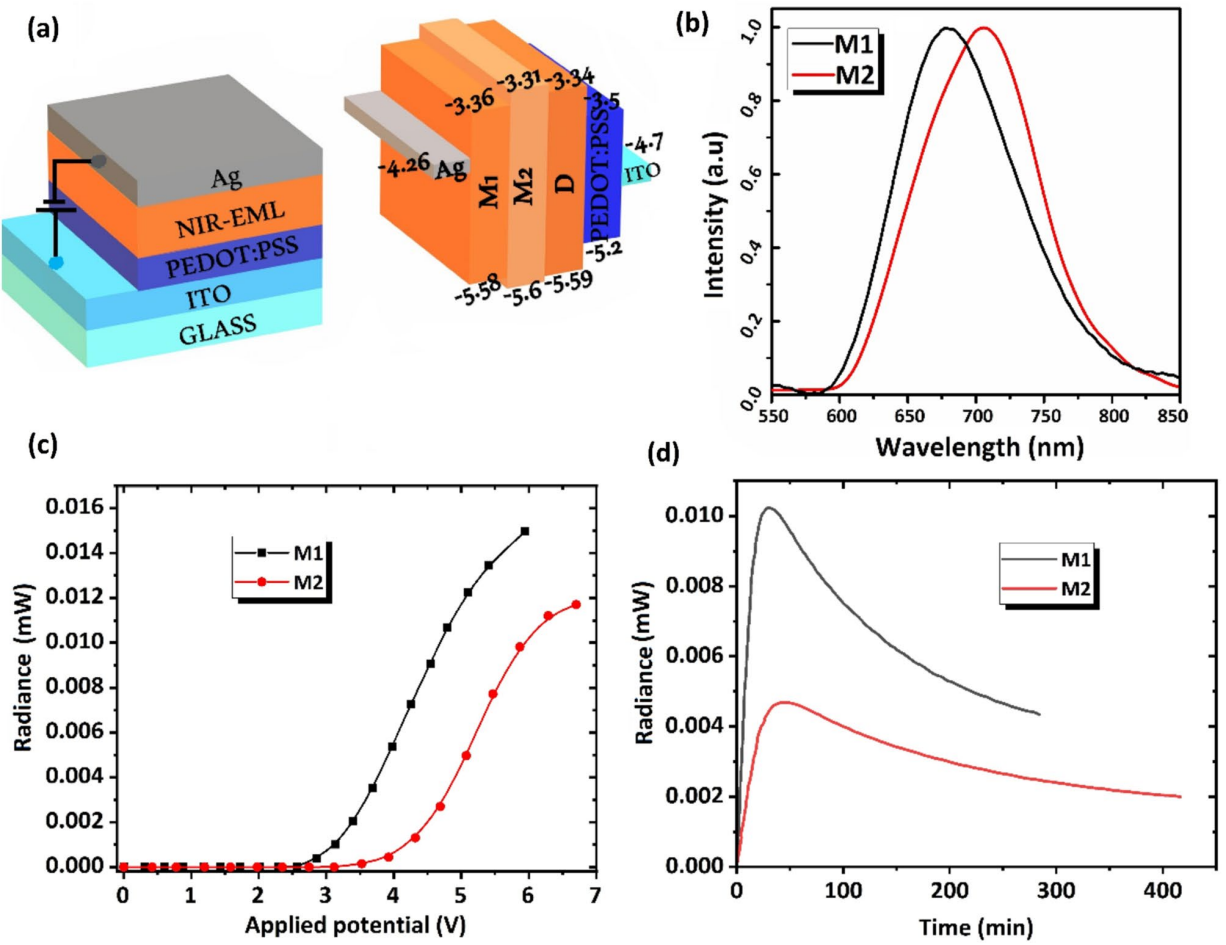
Schematic of the structure of LEC devices and energy level diagrams (**a**), EL spectra (**b**) and radiance vis applied voltage at scan rate of 20 mV/s (**c**) and time (**d**) for M1,2 based LEC devices.

The intense thin film emission along with the reversible redox behavior of these new near-infrared emitters encouraged us to investigate their EL properties in LEC devices. The two-layer LECs with the configuration of ITO/ PEDOT: PSS/iTMC: IL (4:1), /Al (IL: [BMIM^+^(PF_6_)^−^]) were fabricated by spin coating a solution under ambient conditions (Fig. 3a). More details concerning the LEC device fabrication and characterization methods can be found in the ESI. The normalized EL spectra of the illuminated DR to NIR-LECs devices M1 and M2 are shown in Fig. 3b. Compared with [Ru(bpy)_3_](PF_6_)_2_ (see Table S1) M1 and M2-based LECs exhibited significant red-shift electroluminescence with maximum emission peaks at 682 and 703 nm, respectively, in which the electron donation of methyl groups and π-extended structure of bathophen as ancillary ligands, destabilize the HOMO levels and thus the band gaps are reduced (see Table 1). Furthermore, the EL spectra of M1 and M2-based LEC devices were largely red-shifted compared with their PL of solutions, suggesting that the polarity effects of the medium were pronounced in the thin film of emitters, especially for M2 with a red shift of ca. 0.24 eV. Concerning, it has been suggested that probably under high electrical fields in LEC devices, the molecular orbitals of the ionic complex might be polarized and, thus, the energy level would decrease, resulting in the red-shift of the EL spectrum with respect to the PL of the thin film^23^. The radiance (R)–voltage (V) curve of the M1,2-based LECs and their EL data are shown in Fig. 3c, and Table 2. As can be observed, by changing the ancillary ligand from dmbpy to bathophen, a significant influence on the performance of the devices is observed. Due to the lower oxidation potential of M1 compared with M2, the turn-on voltage of 2.7 V was observed for M1-based LEC. In contrast, the M2-based LEC exhibited a higher turn-on voltage of 3.4 V, which can be attributed to the lower oxidation potential and bulky structure of M2 together with the presence of bathophen ancillary ligands. Additionally, the bulky structure of M2 decreased its ionic mobility in the film and thus increased the turn-on time of M2-based LEC (44 min) with respect to M1 (27 min)^24^. Furthermore, as seen in Fig. 3d the M2-based LEC shows a higher lifetime than M1. The stability improvement of M2-based LEC is attributed to the increase of the hydrophobicity of the emissive layer due to the presence of phenyl groups on the bathophen ligands which partly prevents the formation of quencher molecules in the emissive layer under the electric field by hindering the entrance of water molecules^22^. In this regard, a similar result was obtained when bathophen-based Ru(II) and Ir(III) complexes were used as emitters in the LECs^25,26^.

**Table 2:** Optoelectronic characteristics of the LEC devices.

LEC	λ_max, EL_ (nm)	CIE^a^	V_on_^b^	t_1/2_^c^	t_on_^d^	R^e^
M1	682	(0.735, 0.265)	2.7	220	27	0.0118
M2	703	(0.733, 0.266)	3.4	317	44	0.0048

^a^[x, y], Commission International de I’Eclairage 1931 color coordinate.

^b^Turn-on voltage (onset voltage obtained at 2.5 × 10^–4^ mW) (V).

^c^Half-lifetime at 5 V (min).

^d^Turn-on time to reach maximum radiance at 5 V (min).

^e^Radiance at 5 V (mW).

On the contrary, the 2.5 times higher radiance of M1-based LEC than M2 reveals the positive effect of relatively higher electron-donating nature of ancillary ligands on the performance of LECs which is among the highest value for deep-red LECs based on the ruthenium polypyridyl complexes (λ_max_ > 680 nm) reported so far, to the best of our knowledge^12^.

In general, electron-donating groups such as methyl and methoxy groups decrease the band gap of these types of emitters in which facilitate the carrier injection and formation of the p–i–n junction in the emissive layers^27^. On the other hand, in comparison with its analogue Ru(II) complex, complex M2 exhibits a significant red-shifted emission in EL due to the methoxy groups^17^. Moreover, methoxy groups increase the solubility of complexes in polar solvents and facilitate the spin-coating process. Because of the very low intense EL emission of D-based LEC, details on this device are not shown here. Unlike its attractive binuclear structure, it showed a very low LEC device performance. However, in the I-V plot of the devices, there is no sign of D degradation (see Fig. S10, ESI). Accordingly, it has been suggested that there are three reasons for the appearance of the unexpected performance for D-based LEC as follows: (1) Severe self-quenching of emission inducing by adjacent emission centers (see solid PL results). (2) The presence of electronic communication between two different Ru(II) centers that reduces the electroluminescence efficiency^20,28^. (3) The different mobility of anions and cations gives rise to unbalanced charge injection/transport in the recombination zone which increases the quenching of the excitons^29^. It is worth pointing out that during the prototypical synthesis of the binuclear Ru(II) complex the stereoisomers of (Λ, Δ), (Λ, Λ), and (Δ, Δ) are formed as a racemic mixture^30^. It has been shown that the racemic configurations of iTMC emitters illustrate different photophysical properties in the solid state as well diverse LEC features compared with their enantiopure^31^. Although it was not possible to separate the D stereoisomers, the use of pure configurations may lead to better LEC properties.

It is useful to point out that the main goal of this work is not to study the best performance for the DR/NIR LECs. However, our results proved the critical role of various substitutions of emitter molecules (electron-donor and bulky groups) on the performance of DR-LECs. Meanwhile, for the first time, we indicate that the di-nuclearization strategy cannot always be efficient for improving the efficiency of iTMC-LECs, and the molecular engineering of a dinuclear complex is very important in this regard. However, most iTMCs-based DR to NIR-LECs still suffer from low device efficiency due to unfavourable radiative transition at lower emission energies^2,12^. Overall, our findings would benefit for development of highly efficient and stable DR-LEC and other types of light-emitting devices.

## Conclusion

In summary, three novel mono-and binuclear Ru(II) complexes with bulky structures based on phenanthroimidazole ligands were designed and successfully synthesized. All complexes showed intense thin film emission in the deep-red region of the spectrum, as well as reversible redox behaviour. The LEC device based on the mononuclear complex with alkylated ancillary ligand (M1) exhibited higher radiance than others. Moreover, unexpectedly, the binuclear complex showed a significant reduction in the phosphorescence and LEC efficiency compared with its counterpart mononuclear complexes. The results confirm the beneficial potential for fabricating efficient DR to NIR-LECs, using bulky iTMCs with appropriate ancillary ligands. Moreover, this study reveals the significance and the complexity that the metal coordination mode plays on the performance of multinuclear complex-based LECs.

## Supplementary Information

Supplementary Information.

## Data Availability

All data generated or analyzed during this study are included in this published article and its supplementary information file.
