# Author Correction: Unveiling the key role of metal coordination mode and ligand’s side groups on the performance of deep-red light-emitting electrochemical cell

**DOI:** 10.1038/s41598-024-74201-1

**Published:** 2024-10-03

**Authors:** Babak Nemati Bideh, Ahmad Sousaraei, Majid Moghadam

**Affiliations:** 1 Department of Inorganic Chemistry, Faculty of Chemistry and Petroleum Sciences, Bu-Ali Sina University, Hamedan, Iran; 2 Departamento de Química Inorgánica, Facultad de Ciencias, Universidad Autónoma de Madrid, 28049 Madrid, Spain; 3 Department of Chemistry, University of Isfahan, Isfahan, 81746−73441 Iran

Correction to: *Scientific Reports*10.1038/s41598-024-67159-7, published online 11 July 2024

The Acknowledgments section in the original version of this Article was incomplete.

“The authors acknowledge Bu-Ali Sina University for financial support and acknowledge the research council of the University of Isfahan for financial support.”

now reads:

“The authors acknowledge Bu-Ali Sina University for financial support (Grant Number: 1507) and acknowledge the research council of the University of Isfahan for financial support.”

The original Article has been corrected.

