# Supplementary material for: Unveiling the key role of metal coordination mode and ligand's side groups on the performance of deep-red light-emitting electrochemical cell
Source: Sci Rep. 2024 Jul 11;14:16070. doi: 10.1038/s41598-024-67159-7 (PMC11239924; doi:10.1038/s41598-024-67159-7)
Supplement: Supplementary file 1 — Supplementary Information. [file 41598_2024_67159_MOESM1_ESM.docx]

**Supplemenatry Information for**

**Unveiling the key role of metal coordination mode and ligand's side groups on the performance of deep-red light-emitting electrochemical cell**

Babak Nemati Bideh,*^a^ Ahmad Sousaraei ^b^ Majid Moghadam ^c^

^a^ Inorganic Chemistry Department, Faculty of Chemistry and Petroleum Sciences, Bu-Ali Sina University, Hamedan, Iran. Email: b.nemati@basu.ac.ir

^b^ Departamento de Química Inorgánica, Facultad de Ciencias, Universidad Autónoma de Madrid, 28049, Madrid, Spain

^c^ Department of Chemistry, University of Isfahan, Isfahan 81746−73441, Iran

Contents

[1. Materials and Methods 2](#_Toc137042987)

[2. Synthesis and characterization 3](#_Toc137042988)

[3. NMR specrta 5](#_Toc137042989)

[8. Tables 6](#_Toc137042990)

[References: 16](#_Toc137042991)

# 1. Materials and Methods

All reagents and solvents were purchased from commercial sources and used without further purification. NMR spectra were recorded on a Bruker 250 MHz spectrometer with CDCl_3_, D_6_-DMSO and tetramethylsilane (TMS) as solvent and internal reference, respectively. Elemental analyses were performed on Elementar Vario EL CHN elemental analyzer. TOF-Mass were performed on LC-MS/MS "Quattro Micro API micromass Waters 2695. IR spectra were recorded on a Perkin-Elmer 597 spectrometer.The Electrochemical studies of ruthenium complexes (2×10^−3^M) were performed under a dry N_2_ atmosphere at 298 K by using SAMA500 potentiostat electrochemical analyzer with conventional three electrode cell, a Pt disk as the working electrode, a Pt wire as the counter electrode, and Ag/AgCl as the reference electrode. The CV measurements were performed at room temperature using 0.10 M tetrabutylammonium perchlorate (NBu₄ClO_4_) tetrabutylammonium hexafluorophosphate (NBu₄PF₆) as the supporting electrolyte and degassed acetonitrile as the solvent. In CV the following parameters and relation were used: scan rate, 100 mV s^-1^; formal potential E^ο′^ = (E_pa_ +/E_pc_)/2 where E_pa_ and E_pc_ are anodic and cathodic peak potentials, respectively; ∆E_p_ is the peak-to-peak separation. The oxidation (E_ox_) was used to calculate the HOMO/LUMO energy levels and electrochemical energy gap energy (E_gap Elc_) using the equations E_HOMO_=-(E_ox_(vs.F_c_/F_C_^+^)+ 4.8 eV), E_g Elc_ = [E_1/2 ox_ - E_1/2 red_]V and E_LUMO_ = E _HOMO_ + E_g Elc_ eV, which is the half-wave oxidation potential of ferrocene was found to be 0.43 V [1].The optical energy gap (E_g Opt_) was calculated from the intersection of absorption and emission spectra in acetonitrile solution. UV–visible absorption spectra was recorded on an Ultrospec3100 pro spectrophotometer in acetonitrile solutions at 298 K . Photoluminescence (PL) emission spectra of ruthenium complexes in degassed solutions at 298 K was recorded using Varian-Cary Eclipse flourocence spectrophotometer. The PL and PLQYs in neat films were measured with an absolute PLQY measurement system (Hamamatsu C11347) equipped with an integrating sphere. The PLQY (PL quantum yields) were calculated by comparison with [Ru(bpy)_3_]^2+^ in degassed CH_3_CN solution at room temperature as a standard ($\emptyset_{std}=0.095$) [2] using to the well-known following equation:

$$\emptyset_{unk}=\emptyset_{std}.(\frac{I_{unk}/A_{unk}}{A_{std}/I_{std}}).{(\frac{\eta_{unk}}{\eta_{std}})}^{2}$$

In equation,Φ_unk_ is PL quantum yield of ruthenium complexes, I_unk_ and I_std_ are the integrated areas of the corrected PL spectra of the ruthenium complexes and standard respectively, A_unk_ and A_std_ are the absorbances of the ruthenium complexes and the standard at the excitation wavelength (λ_exc_= 450 nm), and η_unk_ and η_std_ are the indexes of refraction of the respective solvents (taken to be equal to the neat solvents in both cases). Neat films of cationic ruthenium complexes:IL (6:1) (thickness of about 160 nm) for study of solid emission were obtained by spin-coating of spectrophotometric grade acetonitrile solution of complexes (5% (w/v)) on a quartz substrate at 800 rpm for 20 s. After evaporation of the solvent in glove-box, the neat films were dried overnight under vacuum at room temperature.

**Device fabrication and measurement :** Indium tin oxide (ITO) coated glass with a sheet resistance of 20 Ω/square and size of 1.5 × 2.0 cm was used as the transparent anode. After being sufficiently cleaned by soaking in ultrasonicated isopropanol, aceton and deionized water, it was dried in the oven at 110°C for 2h. Thin films (ca. 30 nm) of poly(3,4-ethylenedioxythiophene):poly(styrene sulfonate) (PEDOT:PSS) were spin-coated onto ITO/glass substrates at 1300 rpm for 60 s and then baked at 150 °C for 30 min in ambient air. The emissive layers (160 nm) of NIR LECs (iTMC:IL, 4:1, IL: 1-butyl-3-methylimidazolium hexafluorophosphate [BMIM^+(^PF_6_)^-^]) were spin-coated from a 5% (w/v) acetonitrile solution at 800 rpm for 20 s . All solution and film preparation were performed under ambient conditions. After spin coating, the devices have been transferred to an inert atmosphere glove box (<0.1 ppm O_2_ and H_2_O) and heated in 70 ^o^C for 5h to completely removed the solvent. Then, the Ag electrode (70 nm) was thermally vapor deposited and encapsulated into the glove-box. The thicknesses of the films have been measured with an Ambios XP-1 profilometer. All EL measurement were carried out in air atmosphere. The current density, luminescence versus the voltage and emission characteristics of LEC devices were measured using an AvaSpec-125 Fiber Optic spectrophotometer, a SAMA500 electroanalayzer system and a True Color Sensor MAZeT (MTCSiCT Sensor) with a Botest OLT OLED Lifetime-Test System.

# 2. Synthesis and characterization


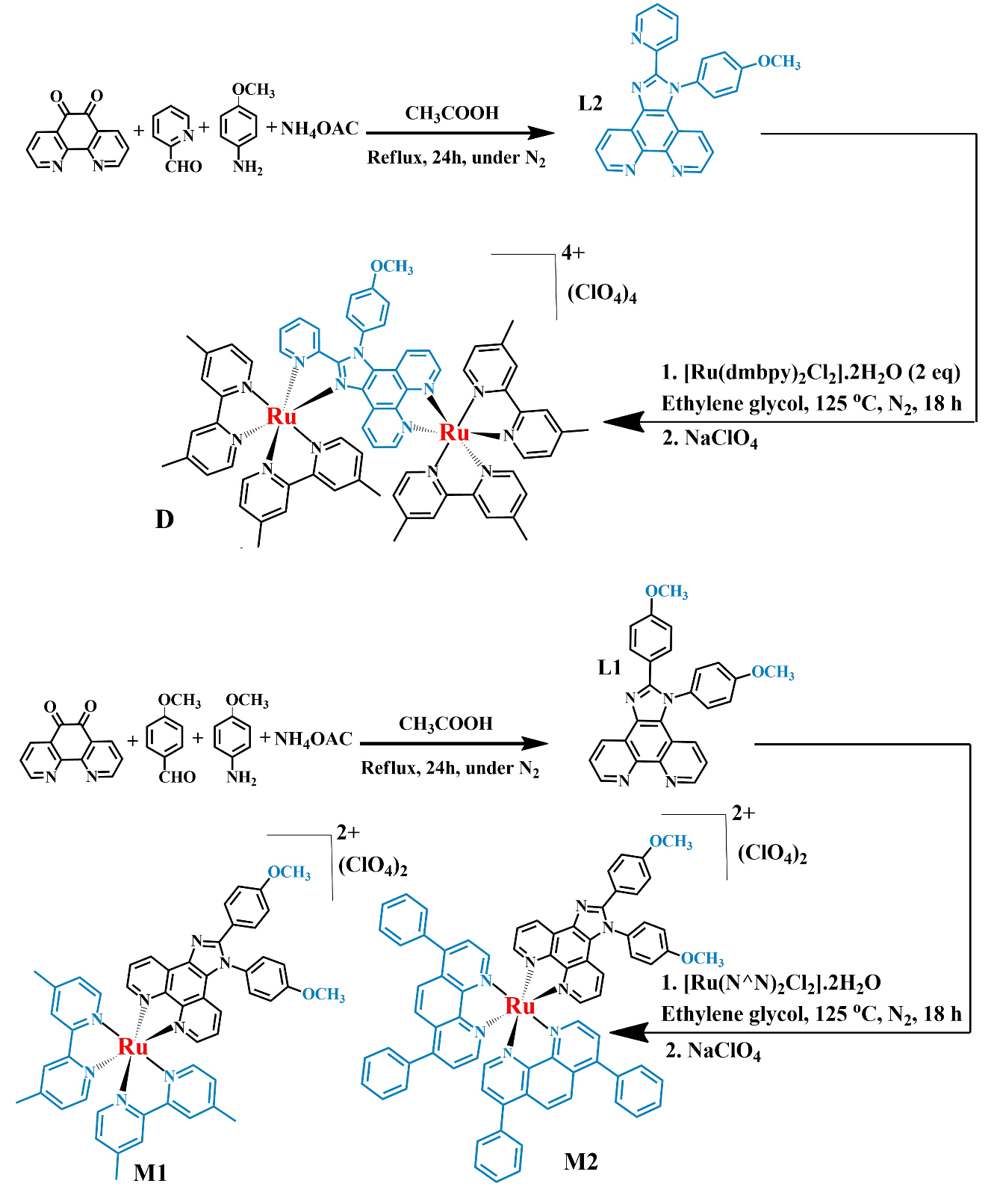


**Scheme S1.** Synthesis of mono and bidentate phenanthroimidazole ligands (L1 and L2) and their ruthenium(II) complexes.

**2.1. Synthesis of precursor materials:**

1,10-phenanthroline-5,6-dione (phendione) and precursor complexes of cis-[Ru(bathophen)_2_Cl_2_].2H_2_O and cis [Ru(dmbpy)_2_Cl_2_].2H_2_O, were synthesized according to the literature reports [3a-d]. 1,2-bis(4-methoxyphenyl)-1H-imidazo[4,5-f][1,10]phenanthroline (L1) ligand was synthesized, characterized, and approved according to the our previous work [3e].

**Synthesis of 1-(4-methoxyphenyl)-2-(pyridin-2-yl)-1H-imidazo[4,5-f][1,10]phenanthroline (L2):**

2-Pyridinecarboxaldehyde (1 mmol, 0.109 gr) and p-anisidine (1.1 mmol , 0.136 g) were dissolved in 5 mL glacial acetic acid and stirred under atmosphere N_2_ for 30 min at room temperature to give a yellow solution. 1,10 phenanthroline-5,6-dione (1mmol) and ammonium acetate (770 mg, 10 mmol, excess) were then added. The reaction mixture was stirred and refluxed at 125 °C under a nitrogen atmosphere for duration of 24 hrs. After this time, the mixture was cooled to room temperature and diluted with 30 mL cool water. The mixture was neutralized with aqueous ammonia solution (25%). The organic compound was extracted with 50 mL of chloroform and dried with Na_2_SO_4_ . The volume of mixture was removed until 1 mml then added to it 15 ml acetone. The clear solution keeps in refrigerator at -5 ^o^C overnight and then the precipitate was isolated. This procedure was repeated one more time to give the product as a white powder. Yield: 42%. mp. 276-279°C, Anal. calcd. For C_25_H_17_N_5_O (%): C, 74.43 ; H, 4.25; N, 17.36. Found (%): C, 74.48; H, 2.25; N, 17.37. IR (KBr): $\tilde{\nu}=$ 3057 (C-H aromatic), 2958 (C-H aliphatic), 1615 (C=C), 1609 (C=N), 1375 cm^-1^ .^1^HNMR (250 MHz, CDCl_3_): 9.15 (m, 2H), 9.04 (d, 1H), 8.43 (d, 1H), 8.00 (d, 1H), 7.68-7.75 (m, 2H), 7.38-7.52 (m, 3H), 7.18-7.33 (m, 2H, overlap with chloroform peak), 7.06 (d, 2H), 3.93 (s, 3H, Hydrogens of methoxy group on N1 imidazole moeity). ^13^CNMR (62 MHz, CDCl_3_): 160.26, 149.10, 149.01, 148.09, 136.30, 131.10, 130.49, 129.68, 128.36, 124.44, 124.07, 123.43, 123.36, 122.13, 120.06, 114.86, 55.58 (Cabon of methoxy group).

**2.2. General procedure for synthesis of mononuclear complexes, M1 and M2, [Ru(N^N)_2_(L1)](ClO_4_)_2_:**

A mixture of 0.1 mmol of cis-[Ru(N^N)_2_Cl_2_].2H_2_O (N^N: dmbpy or bathophen), 0.105 mmol of L1 ligand (42 mg) and 5 ml ethylene glycol was deggased by N_2_ for 30 min and heated under N_2_ atmosphere at 125°C for 18.0 h to give a clear oragnge- red solution. The mixture was cooled to room temperature and then, saturated aqueous solution of NaClO_4_ was added drop by drop to it until gave a orange precipitate and washed several times with distilled water to remove traces of salts. The product was purified by column chromatography on alumina with acetonitril-toluene (3/1, v/v) as an eluent. After the mainly red band was collected, the solvent was removed by rotary evaporation and red solid were obtained.

**CAUTION:** Perchlorate salts of metal complexes with organic ligands are potentially explosive, and only small amounts of the material should be prepared and handled with great care!

**[Ru(dmbpy)_2_(L1)](ClO_4_)_2_ (M1).** Yield: 73 %. IR (KBr): $\tilde{\nu}=$ 3096 (C-H aromatic), 2935 (C-H aliphatic), 1620 (C=C), 1598 (C=N), 1519, 1089 (ClO_4_) cm^-1^. ^1^HNMR (250 MHz, D_6_-DMSO): 9.16 (d, 1H), 8.62-9.03 (m, 3H), 8.11 (d, 1H), 7.88-8.05 (m, 2H), 7.46-7.85 (m, 10H), 7.32-7.44 (m, 2H), 7.09-7.32 (m, 4H), 6.81-7,06 (m, 3H), 3.89 (s, 3H)(hydrogens of methoxy on N1 imidazole moeity of L1), 3.76 (s, 3H) (hydrogens of methoxy on C2 imidazole moeity of L1), 2.52 (12H, Signal of hydrogens of methyl groups on dmbpy are overlapped under DMSO peak). ^13^CNMR (62 MHz, CDCl_3_): 162.07, 160.91, 160.36, 159.44, 158.85, 157.79, 157.08, 156.60, 154.28, 153.33, 150.88, 149.97, 149.40, 145.79, 136.41, 131.11, 130.41, 129.53, 128.95, 127.14, 125.45, 124.55, 121.85, 119.89, 118.71, 118.08, 117.12, 116.25, 114.48, 56.10 (carbon of methoxy group on N1 imidazole moeity of L1), 55.77 (carbon of methoxy group on C2 imidazole moeity of L1), 21.13 (carbon of methyl groups of dmbpy). Anal. calcd. For C_51_H_44_Cl_2_N_8_O_10_Ru (%): C, 55.64; H, 4.03; N, 10.18. Found (%): C, 55.62; H, 4.02; N, 10.19. TOF-MS (m/z): 451.48 ([M1-2ClO_4_]^2+^).

**[Ru(bathophen)_2_(L1)](ClO_4_)_2_(M2).** Yield: 81%. IR (KBr): $\tilde{\nu}=$ 3073 (C-H aromatic), 2957 (C-H aliphatic), 1614 (C=C), 1607 (C=N), 1543, 1088 (ClO_4_) cm^-1^. ^1^HNMR (250 MHz, D_6_-DMSO): 9.23 (d, 1H), 8.12-8.52 (m, 8H), 7.88-8.12 (m, 2H), 7.41-7.81 (m, 31H ), 7.02-7.40 (m, 4H), 3.91 (s, 3H)(hydrogens of methoxy group on N1 imidazole moeity of L1), 3.87 (s, 3H) (hydrogens of methoxy group on C2 imidazole moeity of L1). ^13^CNMR (62 MHz, CDCl_3_): 162.22, 154.51, 154.15, 152.82, 152.53, 151.04, 150.95, 148.65, 148.77, 147.33, 147.15, 138.52, 138.15, 137.65, 131.42, 130.94, 130.38, 130.15, 129.02, 127.82, 127.27, 126.77, 126.45, 126.36, 124.54, 122.38, 116.81, 55.13 (carbon of methoxy group on N1 imidazole moeity of L1), 55.85 (carbon of methoxy group on C2 imidazole moeity of L1). Anal. calcd. For C_71_H_52_Cl_2_N_8_O_10_Ru (%): C, 63.12; H, 3.88; N, 8.31. Found (%): C, 63.14; H, 3.87; N, 8.29. TOF-MS (m/z): 475.87 ([M2-2ClO_4_)]^2+^.

**2.3. Synthesis of dinuclear complex of [Ru_2_(dmbpy)_4_(L2)](ClO_4_)_4_ (D):**

A mixture of 0.2 mmol of cis-[Ru(dmbpy)_2_Cl_2_].2H_2_O and 0.10 mmol of L2 (40 mg) in 8 ml acetonitrile was degassed by nitrogen for 30 min and heated under N_2_ at 125 °C for 18 h. The mixture was cooled to room temperature and then, saturated aqueous solution of NaClO_4_ was added drop by drop to it until gave a orange precipitate. The product was collected by centrifuge and several time washed with distilled water and purified by column chromatography on alumina with acetonitril-toluene (3/1, v/v) as an eluent. Yield: 73%. Anal. calcd. For C_73_H_65_Cl_4_N_13_O_17_Ru_2_ (%): C, 50.38; H, 3.76; N, 10.46, Found (%): C, 50.43; H, 3.73; N, 10.44. ^1^HNMR (250 MHz, D_6_-DMSO): 9.25 (d, 1H), 8.65- 8.93 (m, 10H), 7.76-8.21 (m, 10H), 8.64-8.81 (m, 2H), 7.53-7.71 (m, 3H), 7.39-7.51 (m, 6H), 7.38 (m, 1H), 7.24 (m, 2H), 7.10 (t, 2H), 3.87(s, 3H, Hydrogens of methoxy group of L2), (24H, Signal of hydrogens of methyl groups on dmbpy are buried under DMSO peak). ^13^C NMR (63 MHz, DMSO) δ 162.14, 157.55, 154.76, 152.98, 152.68, 151.46, 150.91, 150.72, 149.89, 148.15, 146.78, 146.06, 137.02, 136.82, 135.58, 130.40, 129.96, 128.89, 128.61, 128.42, 128.36, 127.24, 126.95, 125.85, 125.42, 124.94, 122.85, 117.25, 55.90 (carbon of methoxy group), 21.24 (carbon of methyl group of dmbpy).TOF-MS (m/z): 770.23 ([D-2ClO_4_]^2+^.

# 3. NMR spectra


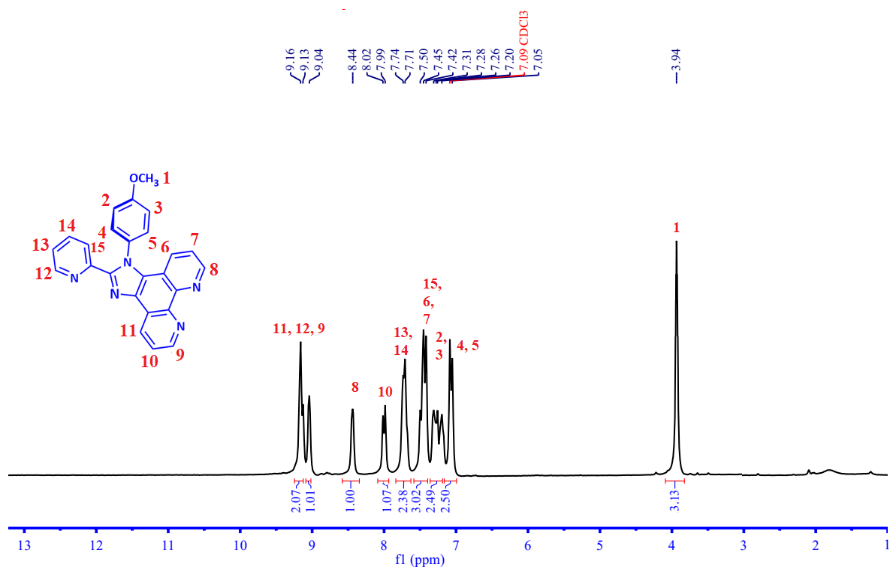


**Figure S1.** ^1^HNMR of L2 in CDCl_3_


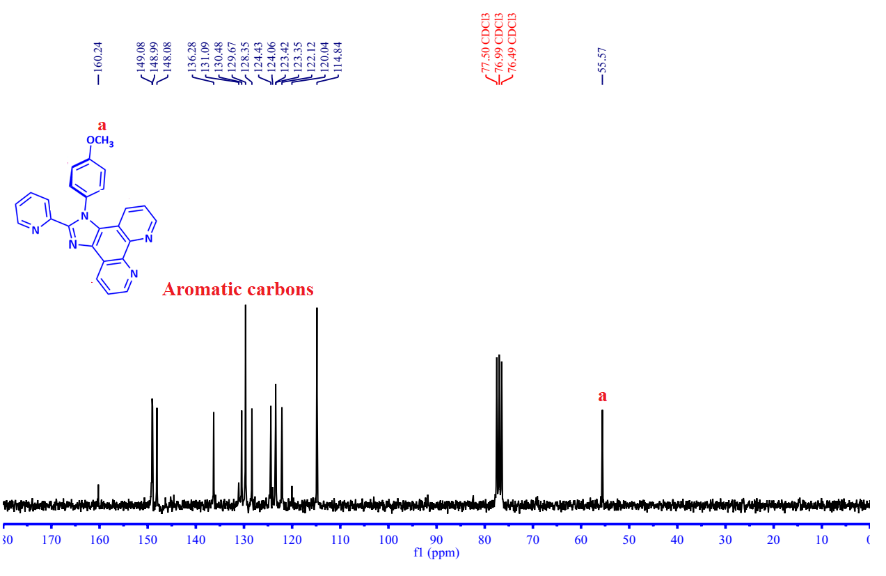


**Figure S2**. ^13^CNMR of L2 in CDCl_3_


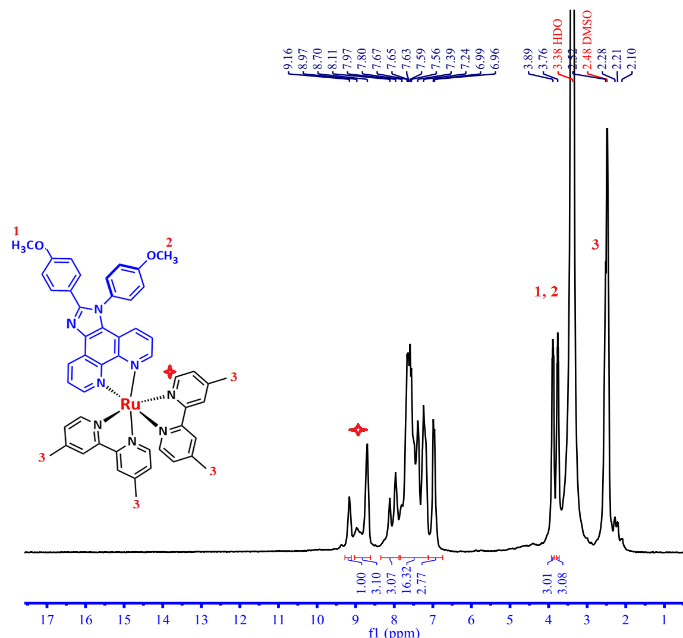


**Figure S3**. ^1^HNMR of M1 complex in D_6_-DMSO


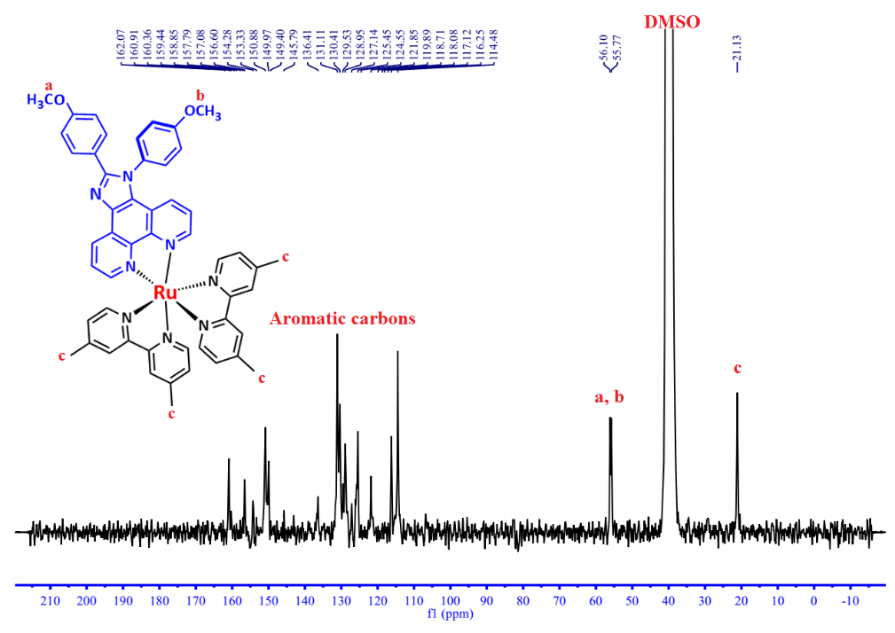


**Figure S4**. ^13^CNMR of M1 complex in D_6_-DMSO.


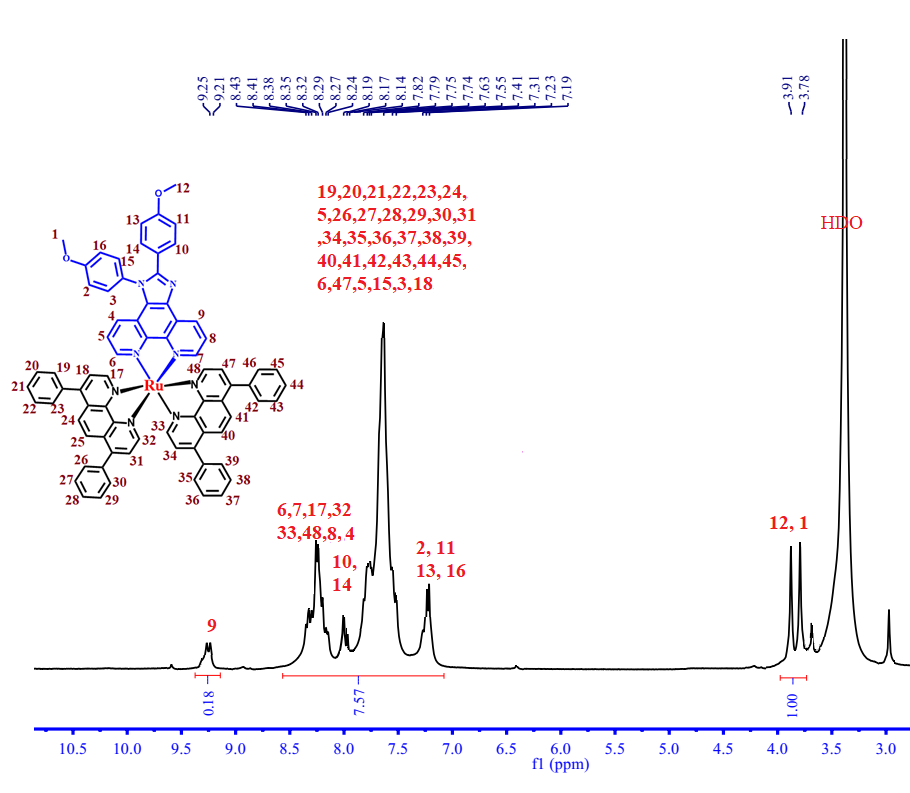


**Figure S5**. ^1^HNMR of M2 complex in D_6_-DMSO


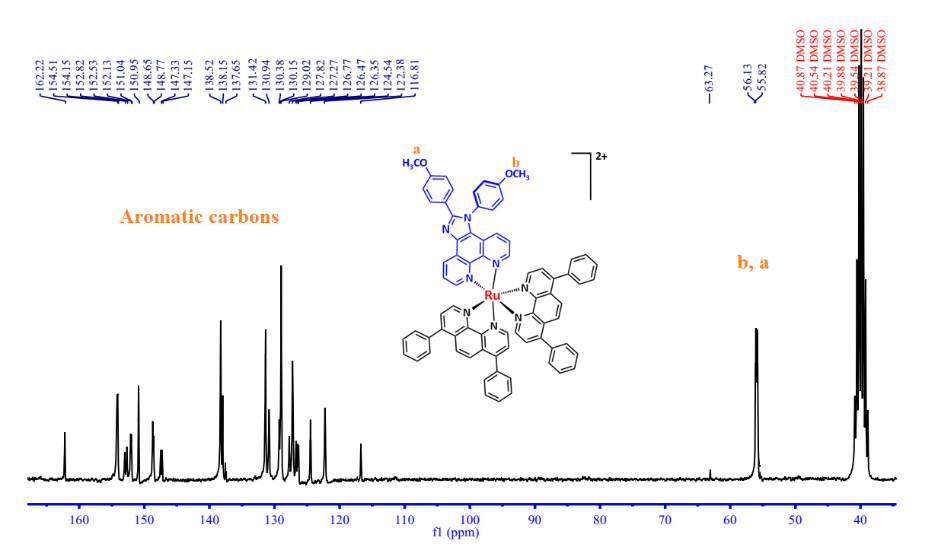


**Figure S6**. ^13^CNMR of M2 complex in D_6_-DMSO


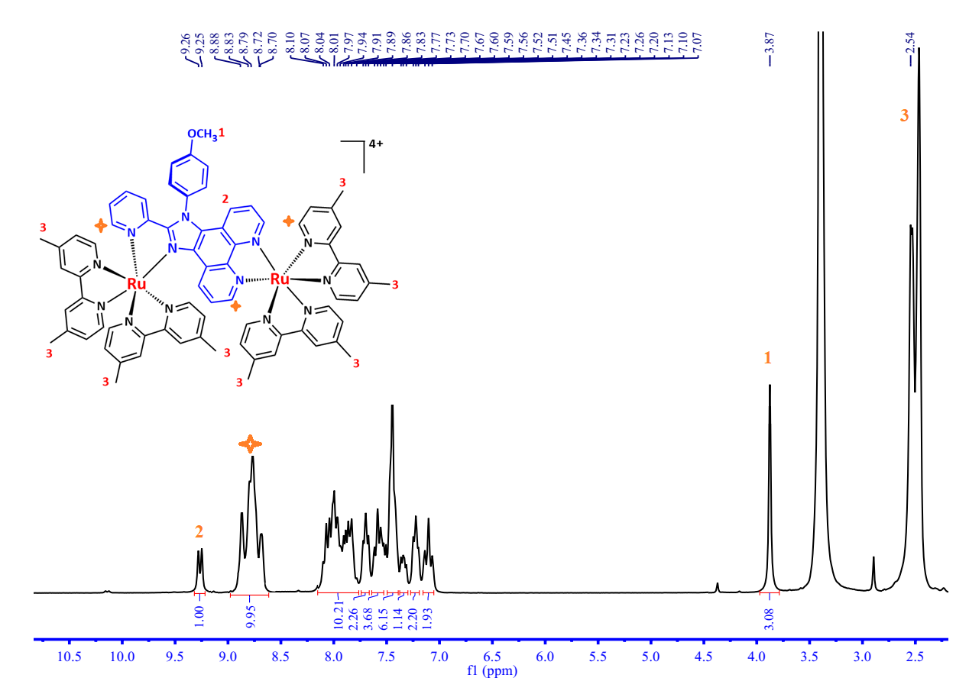


**Figure S7**. ^1^HNMR of D complex in D_6_-DMSO.


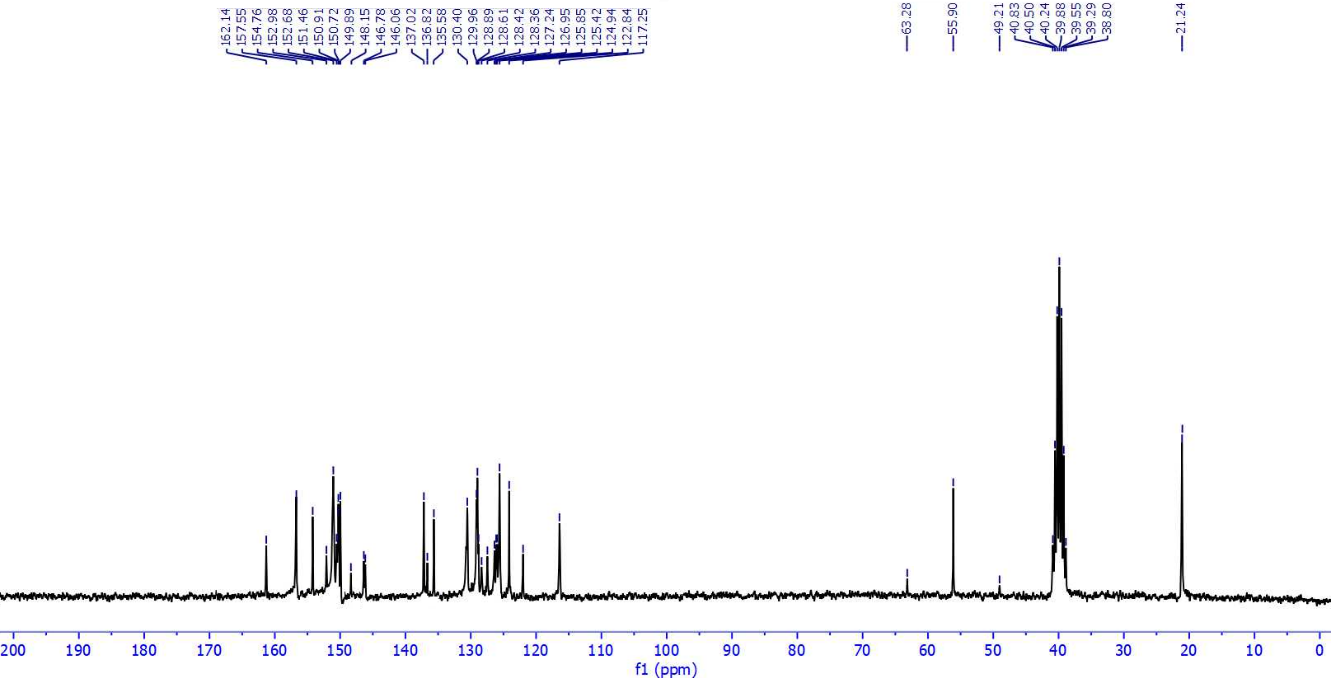


**Figure S8**. ^13^CNMR of D complex in D_6_-DMSO.


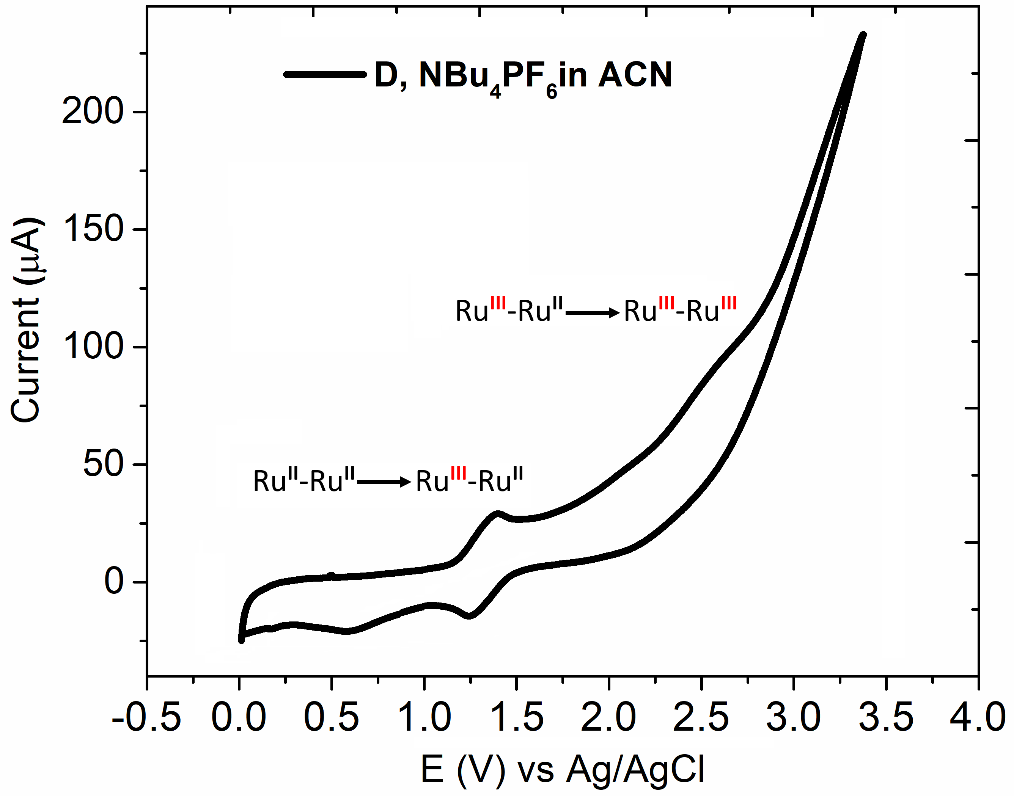


**Figure S9**. Cyclic voltammogram of D in 0.1 M NBu_4_PF_6_ acetonitrile solution (0 - 3.5 V)


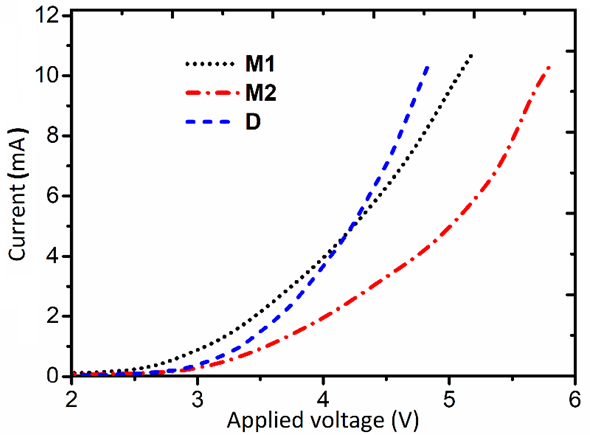


**Figure S10**. Current vs applied voltage for ITO/PEDOT:PSS/Pure M1,2 and D/Ag devices

# 8. Tables

**Table S1**. EL properties of near infrared light electrochemical cell based on **mononuclear ruthenium** polypyridyl complexes

| **Mononuclear ruthenium complexes** | **Cell configuration** | **EL_max_ (nm)** | $\boldsymbol{EQE}$**(%)** | **Ref.** |
| --- | --- | --- | --- | --- |
| 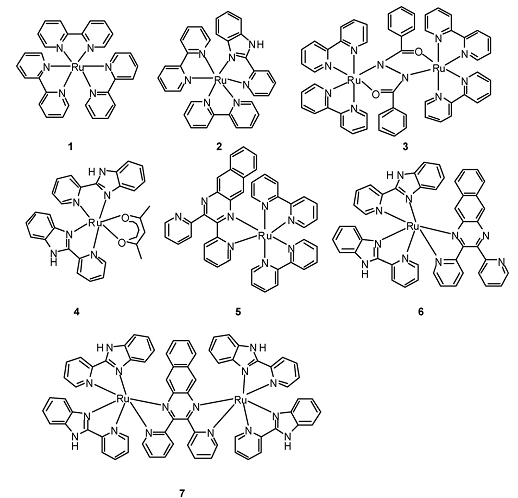 | ITO/complex(90)/ Ga:In | 660 | 1.4 | (4) |
|  | ITO/complex (100 nm)/Au (100 nm) | 630 | 0.31 | (5) |
| 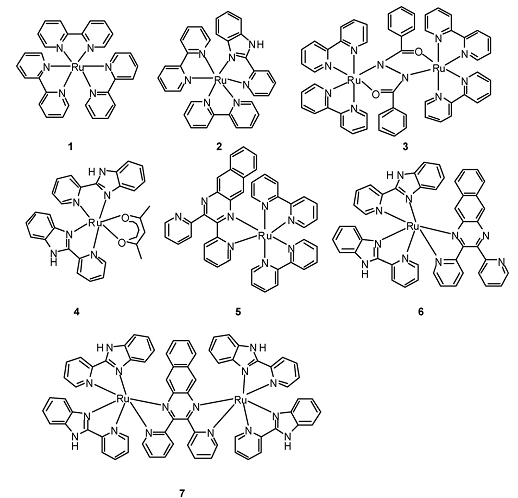 | ITO/complex (100 nm)/Au (100 nm) | 880 | 0.075 | (5) |
| 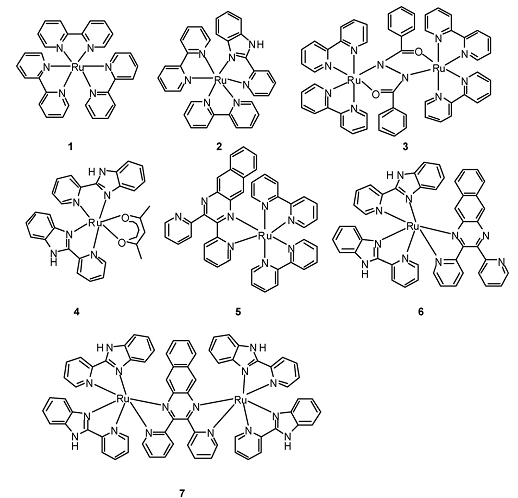 | ITO/complex (100 nm)/Au (100 nm) | 900 | 0.06 | (5) |
| 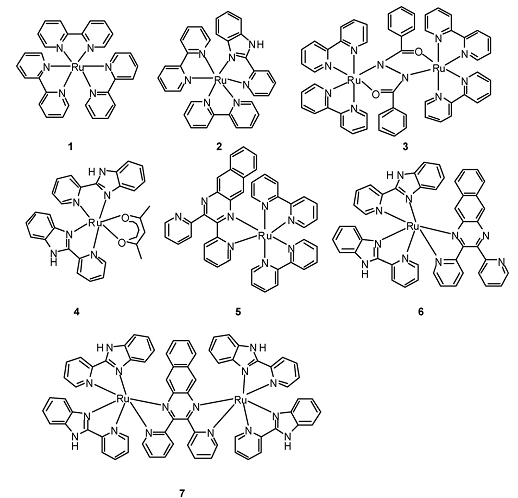 | ITO/complex (100 nm)/Au (100 nm) | 945 | 0.03 | (5) |
| 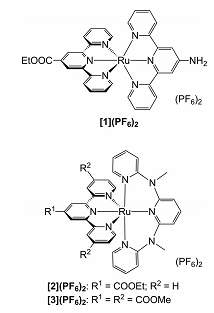 | ITO/PEDOT:PSS(45nm)/Ru:PMMA (169-194 nm)/Ag | 733 | 0.001 | (6) |
| 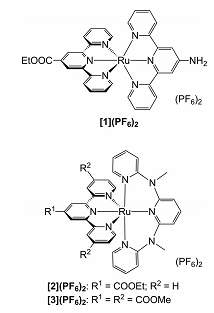 | ITO/PEDOT:PSS(45nm)/Ru:PMMA (169-194 nm)/Ag | 722 | 0.028 | (6) |
| 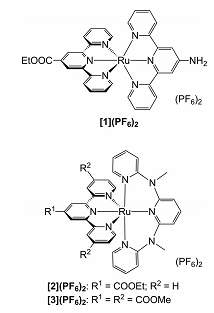  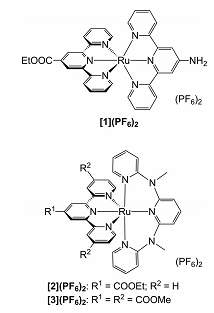 | ITO/PEDOT:PSS(45nm)/Ru:PMMA (169-194 nm)/Ag | 745 | 0.013 | (6) |
|  | ITO/PEDOT:PSS(30nm)/Ru/Al | 600, 720 | 2.06 ,0.27(after 100 min) | (7) |
| 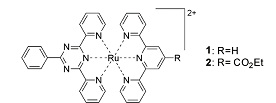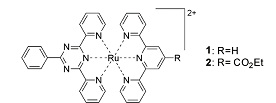 | TO/PEDOT:PSS/ complex:PMMA/Al | 717 | 0.005 | (8) |
| 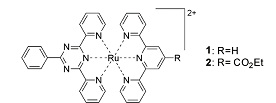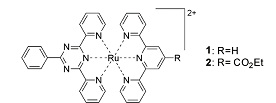 | TO/PEDOT:PSS/ complex:PMMA/Al | 725 | 0.005 | (8) |
| 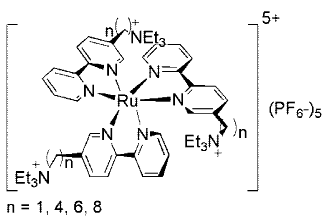 | ITO/[Ru complex]/Au | n=1, 612  n=4, 599  n=6, 599  n=8, 605 | n=1, 0.01  n=4, 0.17  n=6, 0.43  n=8, 0.27 | (9) |
| 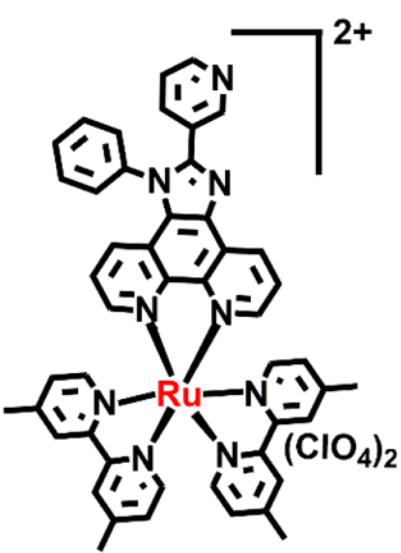 | ITO/complexes (90nm)/ Ga:In | 695 | 0.62 | 12 |
| 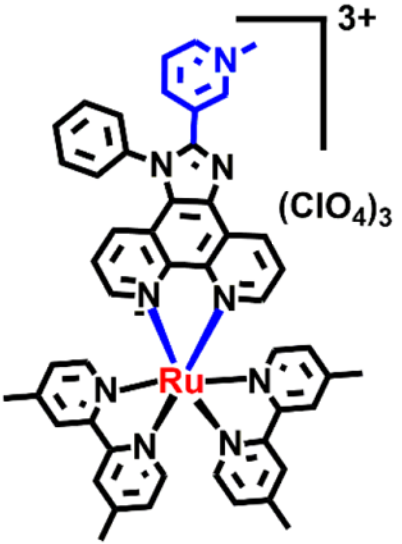 | ITO/complexes (90nm)/ Ga:In | 705 | 0.16 | 12 |

**Table S2**. EL properties of near infrared light electrochemical cell based on **binuclear ruthenium** polypyridyl

| **Mononuclear ruthenium complexes** | **Cell configuration** | **EL_max_ (nm)** | $\boldsymbol{EQE}$**(%)** | **Ref.** |
| --- | --- | --- | --- | --- |
| 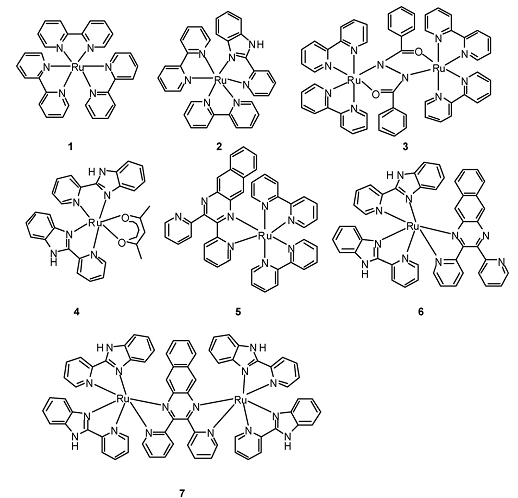 | ITO/complex (100 nm)/Au (100 nm) | 780 | 0.013 | (5) |
| 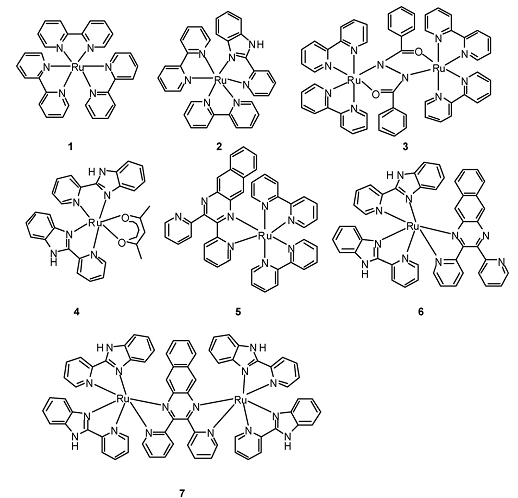 | ITO/complex (100 nm)/Au (100 nm) | 1040 | - | (5) |
| 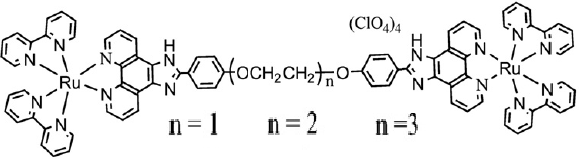 | ITO/complex(90)/Ga:In | n=1, 638  n=2, 626  n=3, 611 | - | (10a) |
| 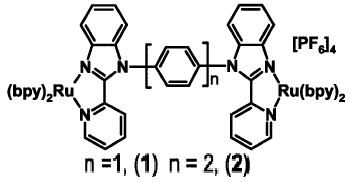 | ITO/complex /Al(100 nm) | n=1, 637  n=2,  657 | - | (1) |
| 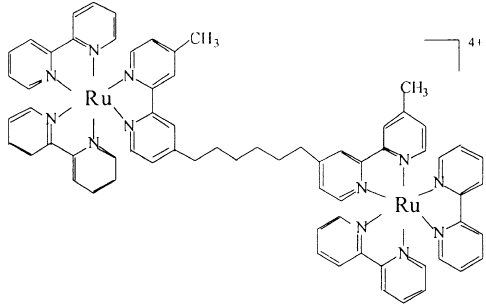 | ITO/ Ru/ Li-triflate /Cr | 638 | 0.02 (at 4V) | (10b) |
| 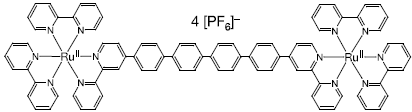 | ITO/Ru:PPV (1:5) /Al(100 nm) | ≈630 (at 4V)  ≈530  (at -4V) | - | (10c) |
| 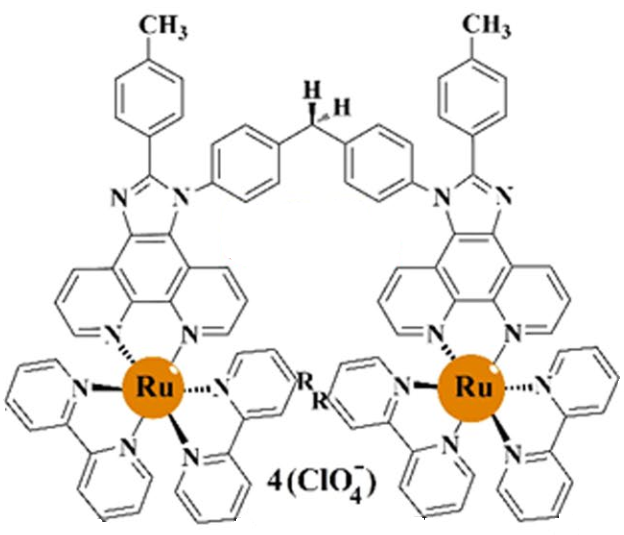 | ITO/complexes (90nm)/ Ga:In | 635 | 0.141 (at 7.5 V) | 11 |
| 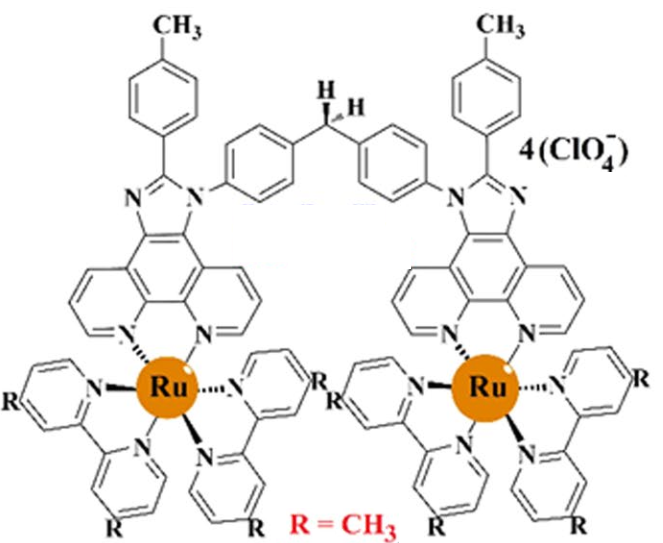 |  | 690 | 0.68 (at 5.9 V) | 11 |

# References:

1. W.-L. Jia, Y.-F. Hu, J. Gao and S. Wang, Linear and star-shaped polynuclear Ru(ii) complexes of 2-(2′-pyridyl)benzimidazolyl derivatives: syntheses, photophysical properties and red light-emitting devices *Dalton Transactions*, 2006, 1721-1728.

2. K. Suzuki, A. Kobayashi, S. Kaneko, K. Takehira, T. Yoshihara, H. Ishida, Y. Shiina, S. Oishi and S. Tobita, Reevaluation of absolute luminescence quantum yields of standard solutions using a spectrometer with an integrating sphere and a back-thinned CCD detector *Physical Chemistry Chemical Physics*, 2009, **11**, 9850-9860.

3. (a) B. Sullivan, D. Salmon and T. Meyer, Mixed phosphine 2, 2'-bipyridine complexes of ruthenium *Inorganic chemistry*, 1978, **17**, 3334-3341. (b) J. Collin and J. P. Sauvage, Synthesis and study of mononuclear ruthenium(II) complexes of sterically hindering diimine chelates. Implications for the catalytic oxidation of water to molecular oxygen, *Inorganic Chemistry*, 1986, **25**, 135-141.(c) R. Caspar, C. Cordier, J. B. Waern, C. Guyard-Duhayon, M. Gruselle, P. Le Floch and H. Amouri, A New Family of Mono- and Dicarboxylic Ruthenium Complexes [Ru(DIP)2(L2)]2+ (DIP = 4,7-diphenyl-1,10-phenanthroline):  Synthesis, Solution Behavior, and X-ray Molecular Structure of trans-[Ru(DIP)_2_(MeOH)_2_][OTf]_2_, *Inorganic chemistry*, 2006, **45**, 4071-4078.(d) W. Paw and R. Eisenberg, Synthesis, Characterization, and Spectroscopy of Dipyridocatecholate Complexes of Platinum, *Inorganic chemistry*, 1997, **36**, 2287-2293. (e) Nemati Bideh, B., Moghadam, M., Sousaraei, Shahpoori Arani, B. [Phenanthroimidazole as molecularly engineered switch for efficient and highly long-lived light-emitting electrochemical cell](https://www.nature.com/articles/s41598-023-29527-7), Scientific Reports, 2023, **13**, 2287.

4. F. G. Gao and A. J. Bard, High-Brightness and Low-Voltage Light-Emitting Devices Based on Trischelated Ruthenium(II) and Tris(2,2‘-bipyridine)osmium(II) Emitter Layers and Low Melting Point Alloy Cathode Contacts, *Chemistry of materials*, 2002, **14**, 3465-3470.

5. S. Xun, J. Zhang, X. Li, D. Ma and Z. Y. Wang, Synthesis and near-infrared luminescent properties of some ruthenium complexes, *Synthetic metals*, 2008, **158**, 484-488.

6. A. Breivogel, M. Park, D. Lee, S. Klassen, A. Kühnle, C. Lee, K. Char and K. Heinze, *European Journal of Inorganic Chemistry*, 2014, **2014**, 288-295.

7. J.-H. Hsu and H.-C. Su, Host-only solid-state near-infrared light-emitting electrochemical cells based on interferometric spectral tailoring, *Physical Chemistry Chemical Physics*, 2016, **18**, 5034-5039.

8. H. J. Bolink, E. Coronado, R. n. D. Costa, P. Gaviña, E. Ortí and S. Tatay, Deep-red-emitting electrochemical cells based on heteroleptic bis-chelated ruthenium (II) complexes, *Inorganic chemistry*, 2009, **48**, 3907-3909.

9. E. Zysman-Colman, J. D. Slinker, J. B. Parker, G. G. Malliaras and S. Bernhard, Improved turn-on times of light-emitting electrochemical cells, *Chemistry of materials*, 2008, **20**, 388-396.

10. (a) C.-C. Ju, C.-H. Chen, C.-L. Yuan and K.-Z. Wang, Electroluminescence from single-layer thin-film devices based on three binuclear Ru (II) complexes with different length of flexible bridges, *Thin solid films*, 2011, **519**, 3883-3889.(b) J.-C. Leprêtre, A. Deronzier and O. Stéphan, Light-emitting electrochemical cells based on ruthenium (II) using crown ether as solid electrolyte, *Synthetic metals*, 2002, **131**, 175-183.(c) S. Welter, K. Brunner, J. Hofstraat and L. De Cola, Electroluminescent device with reversible switching between red and green emission, *Nature*, 2003, **421**, 54-57.

11. B. N. Bideh and H. Shahroosvand, New molecularly engineered binuclear ruthenium (II) complexes for highly efficient near-infrared light-emitting electrochemical cells (NIR-LECs), *Scientific reports*, 2017, **7**, 1-7.

12. B. Nemati Bideh, H. Shahroosvand and M. K. Nazeeruddin, High-Efficiency Deep-Red Light-Emitting Electrochemical Cell Based on a Trinuclear Ruthenium (II)–Silver (I) Complex, *Inorganic Chemistry*, 2021, **60**, 11915-11922.
